# Polypharmacy appropriateness in Italian Long-Term Care Facilities: the nationwide prescription day point survey

**DOI:** 10.1007/s40520-025-03183-5

**Published:** 2025-10-11

**Authors:** Alba Malara, Alberto Zucchelli, Caterina Trevisan, Gilda Borselli, Raffaele Antonelli Incalzi, Graziano Onder, Antonio Cherubini, Alessandra Marengoni, Alessandro Morandi, Anna Castaldo, Dario Leosco, Giuseppe Dario Testa, Andrea Ungar, Prescription Day LTCFs Workgroup

**Affiliations:** 1 Società Italiana di Gerontologia e Geriatria, Via G.C. Vanini, 5, 50129 Florence, Italy; 2 Fondazione ANASTE Humanitas, Via dei Gracchi, 137, 00192 Rome, Italy; 3 Università degli Studi di Brescia, Viale Europa 11, 25123 Brescia, Italy; 4 Aging Research Center, Department of Neurobiology, Care Sciences and Society, Karolinska Institutet and Stockholm University, Stockholm, Sweden; 5 Università degli Studi di Ferrara, Via Ludovico Ariosto, 35, 44121 Ferrara, Italy; 6 Università Campus Bio-Medico di Roma, Via Alvaro del Portilio, 200, 00128 Rome, Trigoria Italy; 7 Università Cattolica del Sacro Cuore, Largo Francesco Vito,1, 00168 Rome, Italy; 8 Fondazione Policlinico Gemelli IRCCS, Largo Agostino Gemelli 8, 00168 Rome, Italy; 9 IRCCS INRCA, Via Don Giuseppe Birarelli, 8, 60121 Ancona, Italy; 10 Università Politecnica delle Marche, Via Tronto 10, 60126 Ancona, Italy; 11 Azienda Speciale Cremona Solidale, Via Brescia, 207, 26100 Cremona, Italy; 12 ASST Centro Specialistico Ortopedico Traumatologico Gaetano Pini-CTO, Piazza Cardinale Andrea Ferrari, 1, 20122 Milan, Italy; 13 Università degli Studi di Napoli Federico II, Via Sergio Pansini, 5, 80131 Naples, Italy; 14 Dipartimento di Medicina Sperimentale e Clinica, Università degli Studi di Firenze, Piazza San Marco,4, 50121 Florence, Italy

**Keywords:** Polypharmacy, Long-Term Care Facilities, Medication prescription, Medication administration

## Abstract

Global population aging is increasing the demand for Long-Term Care Facilities to support older adults with complex health needs. The Prescription Day LTCFs project is a national multicenter point-prevalence study, conducted by the Italian Society of Gerontology and Geriatrics in collaboration with the ANASTE Humanitas Foundation, investigated medication prescription patterns and administration practices in 3,400 residents across 82 facilities in Italy. Participants had a mean age of 84.7 years, with a high prevalence of frailty (49.7%) and polypharmacy (84.8% taking five or more medications). Common chronic conditions included hypertension, dementia, and dysphagia. The study highlighted the complexity of pharmacological regimens, emphasizing risks related to potentially inappropriate medications, drug-drug interactions, and frequent modifications of solid oral dosage forms to facilitate administration in residents with swallowing difficulties or cognitive impairment. These complexities contribute to increased nursing workload. Despite advances in deprescribing research, polypharmacy remains highly prevalent, underlining the need for tailored prescribing guidelines. Variability among Long Term Care Facilities reflects differences in organization and regional healthcare frameworks. The findings provide a valuable foundation for developing strategies to optimize medication management, enhance safety, and improve quality of care in Italian Long Term Care Facilities. This study also offers insights to inform healthcare policies and best practices in pharmacological care for older adults in Long Term Care settings. PRE BIO CE n. 0027032 (20/06/2024), National Ethics Committee, Istituto Superiore di Sanità, Rome, Italy.

**Supplementary Information:**

The online version contains supplementary material available at 10.1007/s40520-025-03183-5.

## Background

Worldwide populations are aging, with individuals living longer but often experiencing disabilities and cognitive decline, which can limit their ability to live independently [1]. Consequently, Long-Term Care Facilities (LTCFs) play a vital role in addressing the needs of older adults unable to live alone due to complex health conditions. LTCF residents are generally older and more likely show frailty and multimorbidity than community-dwelling older adults [2].

The health needs of LTCF residents often require complex medication regimens. Up to 74% of LTCF residents take nine or more medications [3], and many requiring medication administration five or more times per day [4]. Complex medication regimens can negatively impact residents by increasing the risk of being prescribed with Potentially Inappropriate Medications (PIM) and experiencing Drug-Drug Interactions (DDIs), both associated with adverse drug reactions (ADRs) leading to hospitalizations, and mortality [3]—particularly among residents with neurocognitive disorders [5]. Additionally, the risk of DDIs is increased by the involvement of multiple healthcare providers [6] whereas frequent medication administration times reduce medication adherence and compliance among older adults [2, 7]. Furthermore, in LTCFs it has been reported frequent use of modified Solid Oral Dosage Forms (SODFs), e.g., crushing tablets or opening capsules to mix with food or liquids, to facilitate administration to residents with swallowing difficulties or those refusing medications or requiring enteral feeding [8, 9]. This practice can inadvertently lead to inappropriate medication administration, potentially compromising treatment effectiveness and safety for both residents and care providers [8]. Moreover, covert administering modified SODFs without patient consent raises significant clinical and ethical concerns [9]. Lastly, since LTCF residents depend on nurses for medication administration, complex drug regimens increase nursing workloads [10] and healthcare costs [11].

## Aims

In light of this scenario, the Italian Society of Gerontology and Geriatrics (SIGG), in collaboration with the National Association of Territorial Residences (ANASTE) Humanitas Foundation, conducted the Prescription Day LTCFs 2024, a national multicenter point prevalence study aimed to:

- Investigate medication prescription patterns among LTCF residents.
- Evaluate the prevalence of PIMs and DDIs.
- Identify the prevalence and appropriateness of modified SODF administration.
- Assess medication adherence and compliance among LTCF residents.
- Evaluate the impact of polypharmacy management (e.g., frequency of daily medication administration, modifications to SODFs) on nursing workload.
- Collect evidence to establish specific best-practice for medication administration in LTCFs, integrating Italian general recommendations on handling of solid oral pharmaceutical forms.

This article describes the Prescription Day LTCFs methodology, characteristics of study sample and anticipated outcomes.

## Methods

### Study design

The Prescription Day LTCFs project is a national multicenter point-prevalence study conducted on October 1, 2024 In Italy, long-term care facilities (LTCFs) for older adults are accredited by municipalities and regions and may have different names (e.g., assisted living homes, nursing homes, skilled nursing homes, rehabilitation centers, retirement homes). However, these facilities can be grouped according to the type of services provided, following the classification proposed by *Mattoni del SSN – Mattone 12* of the Italian Ministry of Health [12]. Specifically R1 (*Intensive Residential Care Units*), including skilled nursing homes, provide services to non-self-sufficient patients requiring intensive treatments essential to support vital functions. R2 units (*Extensive Residential Care Units*), including nursing homes, are accredited to provide daily medical and nursing care, functional recovery treatments, intravenous therapies, enteral nutrition, and management of complex clinical conditions. R2D units (*Alzheimer Units*) are specialized nursing home wards dedicated to patients with dementia and associated behavioral or affective disturbances; they are frequently organized as accredited modules within R2 units. R3 units (*Maintenance Residential Care Units*), including assisted living facilities, provide long-term care and maintenance services for non-self-sufficient patients with lower health care needs. Finally, rehabilitation centers are dedicated to non-self-sufficient individuals requiring extensive rehabilitative interventions [13].

The invitation to participate in the project was disseminated through the networks of various scientific societies, and professional organizations across the national territory. This approach aimed to reach a broad and relevant audience, ensuring diverse and representative responses for the study. In the project, 138 facilities initially expressed their willingness to participate, on a voluntary basis; of these, 82 facilities voluntarily confirmed their participation and successfully completed the project.

All residents aged 60 and older in participating facilities between 00:00 and 23:59 on the index day were asked to participate in the study. The absence of signed informed consent by the participant or, if the participant was unable to provide consent due to impaired consciousness or awareness, a lack of signed authorization from a relative, caregiver, or trustee was an exclusion criterion.

### Data collection

Physicians and nurses working in residential care settings were trained online to standardize the use of the tools employed in the project, and too collect data using a structured electronic Case Report Form (e-CRF) accessible via an online Research Electronic Data Capture (REDCap) Application instance hosted by SIGG.

Minimum Core Dataset: A minimum core dataset was defined and required to be collected by all participating. This dataset comprised three sections:

1. **Resident characteristics**: demographic data (age, sex); chronic conditions as reported by physicians based on medical history; functional status measured with Activities of Daily Living (ADL), in which a lower score indicates a worse functional state [14]; nutritional status evaluated using the Mini Nutritional Assessment short form (MNA-sf). According to this scale, residents had a normal nutritional status, were at risk of malnutrition or malnourished on scores ranging from 12 to 14, 8–11, 0–7 points respectively [15]; the presence of dysphagia was recorded, along with the method of diagnosis (clinical evaluation, specialist consultation, or specific diagnostic tests). Additionally, the presence of a nasogastric (NG) tube or percutaneous endoscopic gastrostomy (PEG) was documented at the time of data collection; frailty status assessed via the FRAIL-NH scale that considering 5 items such as fatigue, resistance, ambulation, illness, and loss of weight [16]. Finally, the presence of acute medical conditions (e.g., fever, delirium, suspected urinary tract infection) on the day of the assessment was also recorded.
2. **Medications**: To Investigate medication prescription patterns among LTCF residents, all prescribed medications on the index date were recorded. Drugs were identified using the Anatomical Therapeutic Chemical (ATC) [17] classification codes. Dosage, pharmaceutical form, and administration method were also collected. We investigated the presence of drug-drug interactions (DDIs) and potentially inappropriate medications (PIMs) using decision support tools (e.g., International Consensus List of Potentially Clinically Significant Drug-Drug Interactions in Older People [18–20]. Data on the modifications of SODF (e.g.: tablet crushing or capsule opening, and administration with food or drinks) were collected to evaluate the appropriateness of the administration based on existing guidelines [21, 22]. In assessing medication use, two concepts were evaluated: adherence to drug therapy, assessed directly for cognitively intact residents able to understand and follow prescriptions; and, compliance to medication intake without understanding the rationale, assessed for cognitively impaired residents [23, 24].
3. **Facility characteristics and nursing workload**: type of facility, available resources (e.g., number of beds, type and number of nurses and other healthcare personnel) were collected in a specific e-CRF. Data on working hours related to polypharmacy management were collected. The nurses participating were interviewed on timing of medication administration over 24 h. The nurses’ workload was calculated in relation to the administration of pharmacological therapy to patients during different time slots of the day. The calculation was performed by considering modules of 20 patients. The workload was calculated considering specific time intervals (e.g., before breakfast, during breakfast, middle morning, lunch, afternoon, dinner, evening, night and the drugs as needed) throughout the day. The time required for administering therapy was assessed based on the number of nurses in each time slot. This calculation allowed for the determination of a quantitative workload indicator per nurse, useful for analyzing resource management within the facility.

### Ancillary data collection

In addition to the core set of required variables, ancillary information was collected for residents with dementia. This included diagnostic data based on ICD-10 codes, which provide a standardized system for diagnosing and classifying different types of dementia (e.g., Alzheimer’s disease, vascular dementia, etc.) [25]. The severity of dementia was assessed using the Clinical Dementia Rating Scale (CDR), which evaluates six domains: memory, orientation, judgment and problem-solving, community affairs, home and hobbies, and personal re, classifying severity from no dementia to severe dementia [26]. Furthermore, Behavioral and Psychological Symptoms of Dementia (BPSD) were assessed as ancillary clinical information using the Neuropsychiatric Inventory (NPI). This tool evaluates 12 neuropsychiatric symptom categories commonly observed in individuals with dementia, including depression, agitation, anxiety, hallucinations, and other behavioral symptoms. The final score for each symptom is obtained by multiplying the frequency and severity scores, with a maximum total score of 144, indicating severe behavioral and psychological symptoms [27].

### Data management

Participants were assigned unique alphanumeric identifiers. Data, sourced from medical records and collected by physicians and nurses, were pseudonymized and stored electronically via a REDCap Application. The platform was integrated with the BioPortal Ontology server to maintain updated ATC code listings. Participant identifiers linked to personal data will remain accessible to authorized personnel within the recruiting centres for 12 months, after which data will be fully anonymized and stored indefinitely without identifiers.

### Statistical analysis

In 2023, 12,363 residential facilities were active in Italy [28], with 3,607 specifically dedicated to older adults (2,513 in Northern, 702 in Central, and 424 in Southern Italy) [29]. Overall, 273,833 residents aged ≥ 65 years were living in LTCFs [28]. Assuming this population size in 2024, the minimum sample size required (95% CI, 5% margin of error, 50% population proportion) was 384 individuals, as computed with R (sample size computation performed by R software – version 4.3.3, CRAN ^®^, R Core 2022, Vienna, Austria). Our study included 3,400 residents, thus largely exceeding the minimum required and ensuring national representativeness.

DrugBank database (downloaded on the 16th of October 2024) was used to match ATC codes with unique DrugBank IDs, to identify active principles. This was especially relevant when ATC codes represented drug combinations (e.g.: atorvastatin + ezetimibe). Mixed-effects models were used to calculate pooled estimates for population characteristics: linear mixed models estimated pooled means, and generalized linear mixed models (binomial distribution, logit link) were used for pooled proportions. Recruiting centers were included as random effects, whereas the models’ intercepts were used as pooled estimates. Confidence intervals (95% CI) were calculated using Wald approximation. The intraclass correlation coefficient (ICC), reflecting variance due to center clustering, was calculated from the ratio between the random effects variance and total variance. A higher ICC value indicates a larger variability between centers on a certain variable. Sensitivity analyses were conducted including only centers with at least 30 participants (median number of participants per center). Patterns of missing data for core variables were explored. The characteristics of the participants included in the patterns were compared with those of the participants with full information. The role of center clustering on missing patterns was investigated by calculating the ICC from generalized linear mixed models (binomial, logit link), including pattern belonging as a dependent variable and recruiting center as a random effect. Standardized mean differences (SMD) was calculated for all variables reported in Table 1, and median, first and third quartiles were reported. All analyses were performed using R version 4.4.3 (R Foundation for Statistical Computing, Vienna, Austria).

## Results

The study included 3,400 participants recruited from 82 LTCFs across Italy. The geographical distribution of the involved centers is shown in Supplementary Fig. 1. The participating LTCFs comprehended almost all Italian regions, with most involved residents living in facilities in Lombardy, Calabria, and Emilia-Romagna regions. The number of participants enrolled per center ranged from 1 to 177. The ratio of residents participating in the study to the total number of individuals in the LTCFs on the index date varied between 1.4% and 100%, with a median of 60.9% (interquartile range [IQR]: 32–93.4%). Of the 79 centers that provided information about their structural and organizational characteristics, 18 (22,7%) were assisted living facilities coded R3, and 45 (56,9%0.7%) were nursing homes (coded R2) and skilled nursing homes (coded R1), 3 were an exclusive rehabilitation facility. Thirteen.

(16.4.%) of the participating centers included dementia specialized care units (coded R2D). Considering the composition of the multidisciplinary team working in the included LTCFs, except for nurses and healthcare assistants, the most common professionals were physiotherapists (*n* = 74 LTCFs, 93.7%), educators (*n* = 57, 72.2%), social workers (*n* = 49, 62%), and psychologists (*n* = 46, 58.2%). The most frequent specialists working in the LTCFs were geriatricians (*n* = 31, 44.3%) and general practitioners (*n* = 19, 24.1%); moreover, in 29 facilities (36.7%) a geriatrician served as medical director.

As shown in Table 1, the pooled mean age of participants was 84.7 years (95% CI: 84.0–85.3; ICC: 0.08), and 73.7% were female (95% CI: 71.3–75.9%; ICC: 0.04). A total of 96.2% (95% CI: 94.6–97.3%; ICC: 0.24) of participants had at least one impairment in Activities of Daily Living (ADLs). Frailty—defined as meeting at least eight criteria on the Frail-NH scale—was observed in 49.7% of the sample (95% CI: 44.6–54.9%; ICC: 0.16). The most prevalent conditions were hypertension (55.5%; 95% CI: 51.3–59.7%; ICC: 0.12), dementia (48.2%; 95% CI: 39.6–57.0%; ICC: 0.41), cerebrovascular disease (27.2%; 95% CI: 22.5–32.3%; ICC: 0.24), and diabetes (19.7%; 95% CI: 17.7–21.9%; ICC: 0.05). The mean number of drugs prescribed per resident was 7.7 (95% CI: 7.3–8.2; ICC: 0.24). A total of 84.8% of residents were prescribed at least five drugs (95% CI: 81.3–87.7%; ICC: 0.21), and 24.0% were on an excessive polypharmacy regimen (i.e., 10 or more drugs; 95% CI: 19.8–28.7%; ICC: 0.23). Dysphagia was present in 15.5% of participants (95% CI: 12.3–19.0%; ICC: 0.22), and 1.1% (95% CI: 0.1%-1.9%; ICC: 0.43) had a NG tube or PEG. Results remained consistent in a sensitivity analysis excluding centers with fewer than 30 participants. A sensitivity analysis excluding centers with less than 30 participants showed similar results (Table 2).

Resident characteristics varied by facility type. Rehabilitation residents, from only three centers (48 patients), were younger and mostly recent admissions, with higher rates of reduced food intake and polypharmacy, including the greatest exposure to ≥ 10 medications, though the small sample limits generalizability. Functional disability was widespread across all groups, particularly in R1–R2 and R2D, with an average loss of over four ADLs. Frailty was most frequent in R1–R2 (55% severely frail), while dementia and sensory deficits were highest in R2D. R3 residents displayed an intermediate profile. Overall, Rehabilitation residents featured newly admitted, polypharmacy-exposed residents; R1–R2 had the greatest functional decline and frailty; R2D showed high sensory impairments with lower drug use (Table 3).

A total of 2143 participants (63.0%) had complete information for all core variables. Among the participants with missing data, 19 distinct patterns of missingness were identified. The most prevalent missing data pattern (*N* = 541, ICC = 0.47, median standardized mean difference [SMD] = 0.07) involved missing data on the frailty NH scale. Compared to participants with complete data, those in this group had similar age and sex distributions but exhibited a higher prevalence of disability (≥ 1 ADL impaired: 98.9% vs. 95.2%) and dementia (67.6% vs. 46.3%), and were prescribed fewer drugs on average (7.0 vs. 7.9). The second most prevalent pattern (*N* = 363, ICC = 0.97, median SMD = 0.10) was characterized by missing data on at least one activity of daily living (ADL) variable. Participants in this group were comparable to those with complete data regarding age and sex but had a lower prevalence of vision problems (3.4% vs. 9.3%) and a higher prevalence of depression (28.6% vs. 19.8%). The third pattern (*N* = 136, ICC = 0.99, median SMD = 0.15) involved simultaneous missing data on frailty and ADL variables. Participants in this category did not significantly differ from those with complete data, except for a lower prevalence of vision problems (6.6% vs. 9.3%) (Supplementary Fig. 2). All other missing data patterns each represented less than 2% of participants.

## Discussion

This study describes the methodology and preliminary results of the first nationwide point prevalence study addressing drug prescription practices in older persons living in Italian LTCFs. This initiative involved 3,400 residents across 82 centers, who were prescribed a mean 7.7 drugs on the index day, with almost 85% being exposed to polypharmacy. The analysis of the study population revealed the complex health profile of older individuals in this cohort, with a high prevalence of chronic conditions, dementia, frailty, and polypharmacy. These findings align with existing literature. Pasina et al. [30], in an observational study involving over 2,500 older adults in 27 Italian nursing homes, reported a mean number of prescribed medications ranging between 7.1 and 8.6, depending on the presence of dementia. A French multicenter study reported an average of 8.1 prescribed drugs per resident, with over 85% experiencing polypharmacy [31]. The SHELTER study [32], including over 4,000 residents in eight European countries, found that 50% were exposed to polypharmacy, and 24.3% experienced excessive polypharmacy (≥ 10 drugs). Remarkably, our results remain strikingly similar more than a decade later, despite significant developments in deprescribing research and related clinical tools and guidelines [33–35]. The preliminary results suggest that the complexity and appropriateness of pharmacological regimens in LTCF residents remain critical issues, particularly given the high prevalence of polypharmacy and frailty of this population. Moreover, medical education often emphasizes disease-specific guidelines, overlooking multimorbidity and frailty, which are prevalent conditions among LTCF residents. The poor prognosis of LTCF populations [36, 37] further calls into question the ongoing use of medications aimed at primary prevention or long-term benefit—therapies unlikely to achieve intended outcomes in this setting [38]. Despite this, multiple studies document increased drug prescribing and pharmaceutical expenditure in older adults’ final months of life, highlighting the need for more personalized prescribing practices.

In the study sample, 15.5% of participants were affected by dysphagia, and 1.1% were carriers of a NG tube or PEG. As a result, these individuals require medication manipulation, particularly the alteration of solid oral dosage forms, to ensure safe and effective administration tailored to their specific swallowing difficulties and nutritional needs. Equally significant is the manipulation of solid oral dosage forms—such as tablet crushing or capsule opening—to facilitate administration in residents with swallowing difficulties or cognitive disorders [9]. These modifications, frequently performed without adequate guidance, risk altering drug bioavailability, efficacy, and safety, particularly concerning controlled-release formulations, enteric-coated tablets, and drugs with narrow therapeutic indexes. LTCF care teams often lack standardized guidelines regarding safely manipulating solid oral dosage forms, resulting in significant clinical practice variability and reliance on empirical or incomplete knowledge.

Additionally, an important aspect concerns the adherence/compliance issues, especially in residents living with dementia, who represent almost half of our sample. Persons with dementia frequently experience challenges with compliance due to cognitive impairment or resistance to taking medications; in these cases, modifying drug formulations or covert administration, where medications are hidden in food or drink, is sometimes used to prevent refusal or distress [5].

Our analysis reveals some variability across LTCFs in Italy, as shown by high intraclass correlation coefficients (ICCs) (Table 4). Multiple factors likely contribute to this finding. First, the participating facilities provide different services based on the type of long-term care to which they belong (assisted living facilities or nursing homes or skilled nursing homes etc.). Second, Italian LTCFs may differ in their legal frameworks and administrative models, influencing both service delivery and resident selection, also relating to regional differences in the organization and availability of healthcare services—including hospitals, general practitioners, private care providers, and rehabilitation services. These differences may result in LTCFs serving diverse roles across the country and, consequently, are likely to influence the characteristics of residents [39].

The strengths of the Prescription Day LTCFs 2024 study include its large national sample size, and standardized electronic data collection conducted by trained healthcare professionals. However, some limitations are worth considering. First, the data refers to Italian facilities and therefore cannot be generalized to other countries. Furthermore the facilities participated voluntarily in the study, therefore, the data may not be completely representative of the national panorama. The cross-sectional point-prevalence design captured drug prescriptions on a single day, which may not fully reflect variations in prescribing practices over time. Nonetheless, most medications are likely to be prescribed as long-lasting therapies, and we collected data on acute conditions present on the index day allowing us to understand the likely reason for the use of no chronic therapies. Another limitation is that drug prescription data, although standardized using ATC codes, were manually entered by data collectors rather than automatically obtained from electronic prescription systems. While this approach may introduce some errors, our data align with available Italian literature. Moreover, our data collection enables a complete linkage between resident characteristics and drug prescriptions. Lastly, the observed variability should be considered in future analyses. Methods such as multilevel modeling exist to account for this variability; additionally, we believe this variability accurately reflects the diverse landscape of Italian LTCFs.

## Conclusions

The Prescription Day 2024 study provides a unique opportunity to assess pharmacological therapy within Italian Long-Term Care Facilities (LTCFs). This study will play a crucial role in identifying areas of potential inappropriateness in medications prescribing and administration practices and will contribute to the development of relevant monitoring strategies and intervention approaches. By addressing these issues, the study aims to improve the quality of care in LTCFs and optimize therapeutic outcomes for residents. Additionally, the findings can inform future healthcare policies and guide the implementation of best practices in pharmacological management within these settings.

**Table 1:** Characteristics of the study population (*N* = 3400, in 82 LTCFs). Pooled estimates are mean or proportion, as appropriate

	Pooled estimate (95%CI)	ICC	Missing
Age	84.7 (84.0-85.3)	0.08	0
Sex (Female)	73.7% (71.3%-75.9%)	0.04	7
Time In LTCF (at least 1 year)	71.0% (66.8%-74.9%)	0.16	0
Reduced Food Intake	15.4% (12.6%-18.7%)	0.17	720
Hypoacusia or Deafness	12.9% (10.4%-15.7%)	0.19	0
Hypovisus or Blindness	7.5% (7.5%-7.5%)	0.37	0
ADL disability: Bathing	88.4% (85.5%-90.9%)	0.19	595
ADL disability: Dressing	85.0% (81.7%-87.8%)	0.17	596
ADL disability: Toiletting	87.6% (84.7%-90.0%)	0.16	596
ADL disability: Transferring	75.3% (72.1%-78.3%)	0.08	597
ADL disability: Continence	90.6% (87.6%-92.9%)	0.27	622
ADL disability: Feeding	28.7% (25.1%-32.7%)	0.11	595
Number Of ADLs Lost	4.4 (4.3–4.6)	0.10	626
Lost ≥ 1 ADL	96.2% (94.6%-97.3%)	0.24	626
Frailty-NH Criteria	6.7 (6.4–6.9)	0.10	820
Frailty (≥ 8 Frail-NH Criteria)	49.7% (44.6%-54.9%)	0.16	820
No. Of Unique Drugs	7.7 (7.3–8.2)	0.24	0
≥ 5 Drugs	84.8% (81.3%-87.7%)	0.21	0
≥ 10 Drugs	24% (19.8%-28.7%)	0.23	0
Dysphagia	15.5% (12.3%-19.0%)	0.22	169
PEG or NG tube	1.1% (0.1%-1.9%)	0.43	127
Acute conditions on index day			
-Delirium (Any)	0.4% (0.2%-1.1%)	0.57	0
-Fever	0.7% (0.3%-1.5%)	0.44	0
Chronic conditions			
Urinary Tract Infection	0.6% (0.3%-1.4%)	0.48	0
Dementia	48.2% (39.6%-57%)	0.41	0
Cerebrovascular disease	27.2% (22.5%-32.3%)	0.24	0
Depression	19.1% (16.4%-22.2%)	0.13	0
Diabetes	19.7% (17.7%-21.9%)	0.05	0
Heart Failure	6.3% (6.3%-6.3%)	0.28	0
Chronic Kidney Disease	11.4% (9.3%-13.9%)	0.16	0
Hypertension	55.5% (51.3%-59.7%)	0.12	0
Chronic Liver Disease	2.6% (1.8%-3.8%)	0.23	0

*95%CI = 95% Confidence Interval. ICC = intraclass correlation coefficient. LTCF: Long Term Care Facility. ADL = Activities of Daily Living

**Table 2:** Sensitivity analysis excluding centres with less than 30 participants: characteristics of the study population (*N* = 2780, in 44 LTCFs). Pooled estimates are mean or proportion, as appropriate

	Pooled estimate (95%CI)	ICC	Missing
Age	85.0 (84.2–85.8)	0.08	0
Sex (Female)	74.4% (71.6%-77%)	0.04	6
Time In LTCF (at least 1 year)	71.3% (67.1%-75.2%)	0.1	0
Reduced Food Intake	13.3% (10.5%-16.6%)	0.13	584
Hypoacusia or Deafness	13.1% (10.2%-16.7%)	0.17	0
Hypovisus or Blindness	8.8% (5.7%-13.3%)	0.39	0
ADL disability: Bathing	87.7% (83.9%-90.8%)	0.19	486
ADL disability: Dressing	84.1% (80.2%-87.4%)	0.14	487
ADL disability: Toiletting	86.7% (83%-89.7%)	0.16	487
ADL disability: Transferring	74.0% (70.3%-77.4%)	0.07	487
ADL disability: Continence	90.7% (87.1%-93.4%)	0.24	508
ADL disability: Feeding	27.7% (24.2%-31.4%)	0.06	486
Number Of ADLs Lost	4.4 (4.2–4.6)	0.08	511
Lost ≥ 1 ADL	96.1% (96.1%-96.1%)	0.24	511
Frailty-NH Criteria	6.6 (6.3–6.9)	0.09	659
Frail (≥ 8 Frail-NH Criteria)	49.5% (43.9%-55%)	0.12	659
No. Of Unique Drugs	7.7 (7.2–8.2)	0.22	0
≥ 5 Drugs	83.8% (79.3%-87.5%)	0.21	0
≥ 10 Drugs	24.2% (19%-30.2%)	0.22	0
Dysphagia	14.4% (10.7%-18.6%)	0.20	125
PEG or NG tube	0.1% (0.0%-1.7%)	0.33	97
Acute conditions on index day			
-Delirium (Any)	0.4% (0.2%-1.3%)	0.58	0
-Fever	0.7% (0.3%-1.6%)	0.38	0
-Urinary Tract Infection	0.8% (0.4%-1.6%)	0.44	0
Chronic conditions			
Dementia	51.8% (42.4%-61.1%)	0.33	0
Cerebrovascular disease	27.5% (22.1%-33.6%)	0.21	0
Depression	19.3% (16%-23.2%)	0.13	0
Diabetes	19.6% (17.2%-22.2%)	0.05	0
Heart Failure	5.1% (3.6%-7.4%)	0.27	0
Chronic Kidney Disease	11.8% (9.4%-14.6%)	0.13	0
Hypertension	55% (49.9%-60%)	0.11	0
Chronic Liver Disease	2.9% (1.9%-4.4%)	0.25	0

*95%CI = 95% Confidence Interval. ICC = intraclass correlation coefficient. LTCF: Long Term Care Facilities. ADL = Activities of Daily Living

**Table 3:** Characteristics of the study population by facility type. *N* = 3310 (*N* = 290 excluded due to missing information on the characteristics of the LTCF). Pooled estimates are mean or proportion, as appropriate

	Pooled estimate (95%CI)			
	R3 Units *N* = 702 *N* centres = 18	Rehabilitation Units *N* = 48 *N* centres = 3	R1-R2 Units *N* = 1509 *N* centres = 45	R2-R2D Units *N* = 1051 *N* centres = 13
Age	85.6 (84.3–86.9)	82.7 (80.4–85)	84.5 (83.6–85.4)	84.8 (83.5–86)
Sex (Female)	75.7% (71.1%-79.8%)	70.8% (56.6%-81.9%)	74.2% (70.3%-77.7%)	71.9% (68.3%-75.2%)
Time In LTCF (at least 1 year)	70.8% (61.7%-78.4%)	1.3% (0%-97.9%)	71.8% (67.9%-75.5%)	76.4% (66.7%-84%)
Reduced Food Intake	15% (10.4%-21.1%)	32.1% (12.9%-60.1%)	14.8% (11.2%-19.4%)	13.9% (8.2%-22.7%)
Hypoacusia or Deafness	15.6% (11.3%-21%)	10.1% (3.1%-28.7%)	10.5% (7.4%-14.7%)	18.5% (13.8%-24.4%)
Hypovisus or Blindness	6.6% (4.1%-10.4%)	9% (1.9%-34.3%)	6.3% (3.7%-10.6%)	15.8% (7.9%-29.2%)
ADL disability: Bathing	87.4% (78.7%-92.9%)	86.4% (86.3%-86.5%)	89% (85.6%-91.6%)	88.7% (80.3%-93.8%)
ADL disability: Dressing	81.4% (71.2%-88.6%)	75.4% (48.9%-90.8%)	86.5% (82.5%-89.8%)	85.5% (78.2%-90.6%)
ADL disability: Toiletting	84.6% (74.8%-91%)	72.9% (58.8%-83.6%)	88.4% (84.6%-91.3%)	90.3% (85%-93.9%)
ADL disability: Transferring	69.8% (63.2%-75.7%)	66.7% (52.3%-78.5%)	79.6% (75%-83.4%)	70.9% (64.7%-76.4%)
ADL disability: Continence	84.5% (77%-89.9%)	83.3% (70.1%-91.4%)	91.8% (87.8%-94.6%)	94.7% (89.5%-97.4%)
ADL disability: Feeding	28.6% (22.9%-35.1%)	21.1% (7.9%-45.4%)	29% (23.2%-35.5%)	31.4% (25.3%-38.2%)
Number Of ADLs Lost	4.2 (3.9–4.6)	3.9 (3.2–4.7)	4.5 (4.4–4.7)	4.5 (4.2–4.8)
Lost ≥ 1 ADL	93.8% (87.9%-97%)	96.8% (70.7%-99.7%)	96.2% (94%-97.6%)	97.5% (94.1%-99%)
Frailty-NH Criteria	6.3 (5.6-7)	6.2 (4.7–7.7)	7 (6.7–7.3)	6.3 (5.8–6.8)
Frailty (≥ 8 Frail-NH Criteria)	44.3% (35.3%-53.6%)	37.3% (11%-74.1%)	55.5% (47.9%-62.9%)	44.2% (36.3%-52.3%)
No. Of Unique Drugs	8.3 (7.5–9.1)	10 (7-12.9)	7.6 (7-8.1)	7.1 (6.2–8.1)
≥ 5 Drugs	89.4% (82.6%-93.7%)	93.7% (82.3%-98%)	83.7% (78.9%-87.6%)	79.4% (68.8%-87.1%)
≥ 10 Drugs	30.2% (22.2%-39.7%)	38.4% (14.9%-68.9%)	22.4% (17%-28.9%)	18.1% (10.4%-29.5%)
Acute conditions on index day				
-Delirium (Any)	1.5% (0.5%-4.6%)	-	0.5% (0.1%-1.9%)	0.1% (0%-11.8%)
-Fever	1.1% (0.3%-3.7%)	4.2% (1%-15.2%)	0.6% (0.2%-2%)	0.9% (0.2%-3.8%)
-Urinary Tract Infection	0.4% (0%-5.2%)	-	0.9% (0.3%-2.3%)	0.8% (0.3%-2.2%)
Chronic conditions				
Dementia	47.8% (32.5%-63.5%)	14.8% (14.7%-14.9%)	43.4% (32%-55.6%)	72.7% (56.2%-84.6%)
Cerebrovascular disease	18.9% (13.6%-25.6%)	29.2% (18.1%-43.4%)	30.4% (23.5%-38.3%)	24.7% (15.5%-37.1%)
Depression	19.5% (14.1%-26.3%)	20.2% (9.4%-38.3%)	18.7% (14.6%-23.6%)	18.6% (14.1%-24.1%)
Diabetes	22.3% (17.7%-27.7%)	16.7% (8.6%-29.9%)	19.7% (16.7%-23.1%)	18.5% (15.4%-22%)
Heart Failure	5.2% (2.5%-10.5%)	7.5% (1.9%-25.1%)	8.3% (5.8%-11.7%)	3.8% (2%-7.4%)
Chronic Kidney Disease	12.6% (9%-17.3%)	9.6% (1.3%-47.2%)	9.9% (7.5%-13%)	14% (8.9%-21.3%)
Hypertension	54.4% (47.7%-61%)	64.6% (50.2%-76.7%)	53.9% (46.7%-60.9%)	56.1% (50%-62.1%)
Chronic Liver Disease	1.5% (0.4%-5.1%)	-	3.2% (2.1%-4.8%)	2.7% (1%-6.8%)

**Table 4:** Factors contributing to variability among LTCFs in Italy

Factors of variability between facilities	Description
Type of facility	assisted living facilities (R3), nursing homes (R2), skilled nursing homes (R1), dementia specialized care units (R2D), rehabilitation centers
Different Regional Legal framework	Managed at regional and municipal levels. Varied accreditation criteria for facilities. Different quality standards and operating protocols. Specific rules for dementia care units or specialized services.
Different Regional Management Models	Public, private non-profit, and private for-profit ownership. Different organizational structures (centralized vs. decentralized). Variability in staffing models (number, type, and qualification of staff). Distinct approaches to resource allocation and service integration.
Diferent Impact on Service Delivery	Range and type of services offered differ (basic care, skilled nursing, rehab, dementia care). Variability in care intensity and continuity of care. Differences in access to external healthcare services (hospitals, specialists).

## Supplementary Information

Below is the link to the electronic supplementary material.

Supplementary Material 1

## Data Availability

No datasets were generated or analysed during the current study.
