# Supplementary material for: Polypharmacy appropriateness in Italian Long-Term Care Facilities: the nationwide prescription day point survey
Source: Aging Clin Exp Res. 2025 Oct 11;37(1):291. doi: 10.1007/s40520-025-03183-5 (PMC12515235; doi:10.1007/s40520-025-03183-5)
Supplement: Supplementary file 1 — Supplementary Material 1 [file 40520_2025_3183_MOESM1_ESM.docx]

**Supplementary Fig. 1.** Geographic distribution of the frequency of long-term care facilities residents involved in the Prescription Day LTCFs 2024


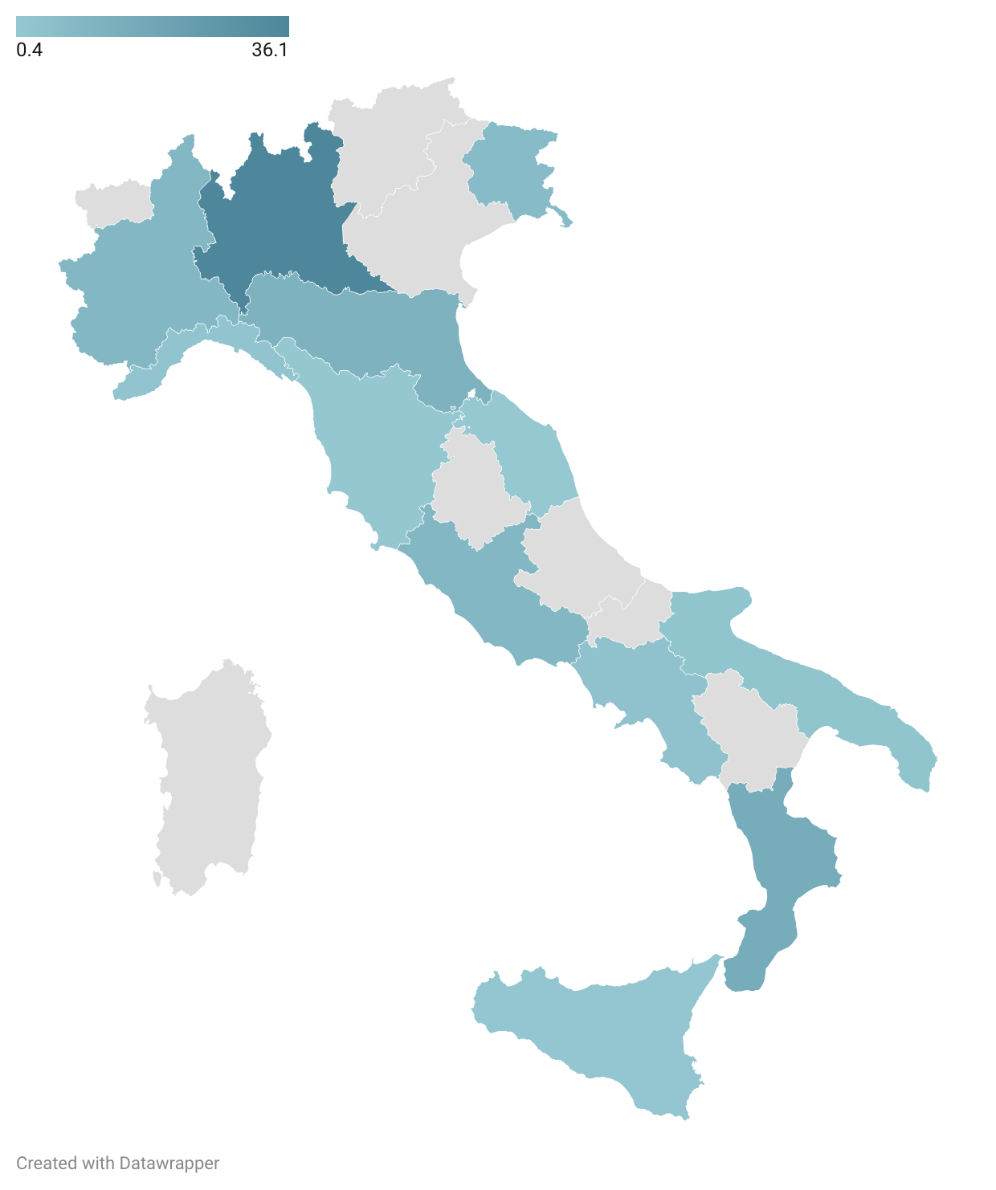


*Notes*. Blue color intensity corresponds to the frequency of residents involved in the study across Italian regions. Grey areas indicate regions with no participating facilities.

**Supplementary Fig. 2**: patterns of missing data in participants without dementia. Prevalence is calculated among participants without dementia. Only patterns with a prevalence ≥ 2.0% are shown. Red = missing data.

| **Pattern n.** | **N (%)** | **Median SMD  (IQR)** | **Demographics** | | **ADLs** | **Frailty NH** | **Acute Conditions** | **Adherence** | **Compliance** | **Dysphagia presence/diagnosis** | **PEG tube or NG tube** |
| --- | --- | --- | --- | --- | --- | --- | --- | --- | --- | --- | --- |
| 1 | 541 (15.9) | 0.07  (0.03-0.21) |  | |  |  |  |  |  |  |  |
| 2 | 363 (10.7) | 0.11 (0.06-0.25) |  | |  |  |  |  |  |  |  |
| 3 | 136 (4.0) | 0.15 (0.06-0.24) |  | |  |  |  |  |  |  |  |
| TOTAL MISSING VALUES: | | | | 23 | 626 | 820 | 0 | 0 | 0 | 169 | 127 |

SMD: standardized mean difference; ADL: activities of daily living; Frailty NH: frailty nursing home scale; PEG: percutaneous endoscopic gastrostomy, NG: naso-gastric
